# Effects of Laparoscopic Roux-en-Y Gastric Bypass for Type 2 Diabetes Mellitus: Comparison of BMI > 30 and < 30 kg/m^2^

**DOI:** 10.1007/s11695-017-2926-9

**Published:** 2017-09-13

**Authors:** Zhigang Ke, Fan Li, Jing Chen, Yu Gao, Xunmei Zhou, Fang Sun, Chunxue Li, Baohua Liu, Qiang Li, Zhiming Zhu, Weidong Tong

**Affiliations:** 1 Department of General Surgery, Institute of Surgery Research, Daping Hospital, Third Military Medical University, Chongqing, 400042 China; 2 Center of Hypertension and Metabolic Diseases, Department of Hypertension and Endocrinology, Daping Hospital, Third Military Medical University, Chongqing, 400042 China

**Keywords:** Metabolic surgery, Type 2 diabetes mellitus, Roux-en-Y gastric bypass

## Abstract

**Background:**

Recently, many studies focused on type 2 diabetes mellitus (T2DM) patients with body mass index (BMI) < 30 kg/m^2^ and suggested that those patients might benefit from Roux-en-Y gastric bypass (RYGB). However, evidence on its effectiveness to improve T2DM patients with BMI < 30 kg/m^2^ is still lacking. The aim of this study is to explore whether T2DM patients with BMI < 30 kg/m^2^ get similar surgical effect from RYGB compared with those patients with BMI > 30 kg/m^2^.

**Methodology:**

Seventy patients with uncontrolled T2DM underwent laparoscopic RYGB from May 2010 to December 2015 in the GI Department of Daping Hospital. Weight, BMI, waist circumference, glucose, and lipid metabolic parameters were collected and evaluated at baseline and 1, 3, 6, 12, and 24 months postsurgery. Patients with BMI < 30 kg/m^2^ were compared with those with BMI > 30 kg/m^2^.

**Results:**

Among the 70 patients, 47 (67.1%) BMI < 30 kg/m^2^, and 23 (32.9%) BMI > 30 kg/m^2^. Patients with BMI < 30 kg/m^2^ are significantly older; they are female predominant and have longer duration of diabetes. The complete remission of T2DM was 28.2% of the BMI < 30 kg/m^2^ group and 57.9% of the BMI > 30 kg/m^2^ group (*p* = 0.029). There was no significant difference in the change of glucose and lipid metabolic parameters of both groups. FPG, 2hPG, and HbA1c% levels were significantly improved after 1 month (*p* < 0.05), and then remained essentially stable from the sixth month in both groups.

**Conclusions:**

The 2-year study has shown that RYGB is a safe and effective procedure in treating T2DM with BMI < 30 kg/m^2^, although the complete remission of T2DM in the BMI < 30 kg/m^2^ group is lower than the BMI > 30 kg/m^2^ group.

## Introduction

Bariatric surgery has been demonstrated to be very successful in treating obese patients with T2DM [1–3] and those with microvascular and macrovascular complications [4, 5]. Roux-en-Y gastric bypass (RYGB) is the most commonly performed procedure worldwide, which has been shown to result in significant weight loss and resolution of T2DM in morbid obese patients [6]. Recently, the role of metabolic surgery has been explored as the treatment of T2DM patients with low body mass index [7–10]. Particularly in China, the mean BMI of T2DM patients is just 24 kg/m^2^ [11]; surgical treatments have become more and more important as therapies for non-obese T2DM patients [12–15]. Therefore, in this study, we investigated the role of RYGB in treating non-obese Chinese T2DM patients with BMI < 30 kg/m^2^. We specifically evaluated the safety and efficacy in those with a BMI < 30 kg/m^2^ and compared it with those with BMI > 30 kg/m^2^. Additionally, we evaluated the effect of RYGB on lipid disorders.

## Materials and Methods

### Ethical Considerations

The study protocol was approved by the Ethics Committee and institutional review at our hospitals and was compliant with the Helsinki Declaration. Each patient provided a signed informed consent after being made aware of the current standards of treatment for T2DM and understanding the risks and benefits associated with the procedure.

### Patients

Seventy patients with uncontrolled T2DM received RYGB at Daping Hospital during the period of May 2010 to December 2015; all the patients are Asiatic populations. The inclusion criteria were as follows: diagnosis of T2DM or other important co-morbidity based on the criteria of the American Diabetes Association (ADA) [16], from 18 to 60 years of age, BMI > 25 kg/m^2^ (based on China’s obesity and type 2 diabetes surgical treatment guidelines (2014)). A patient would be excluded if he or she had previously undergone bariatric surgery or other complex abdominal surgery, as were those with established diagnoses of type 1 diabetes, latent adult autoimmune diabetes, malignancy, pregnancy, neurologic disease, or cardiovascular disease.

Prior to the operation, each patient was assessed by a multidisciplinary team (MDT) comprised of a surgeon, endocrinologist, anesthetist, psychiatrist, and dietician. Moreover, each patient underwent a routine preoperative workup and counseling in addition to a detailed diabetic workup. The preoperative and postoperative data sets were collected and entered into a database. Preoperatively, we collected data on patient demographics, height, weight, BMI, waist circumference, co-morbidity, duration of T2DM, medication use, fasting plasma glucose (FPG), 2-hour postprandial glucose (2hPG), and glycosylated hemoglobin (HbA1c). Additionally, the levels of total cholesterol (TC), triglyceride (TG), low-density lipoprotein (LDL), high-density lipoprotein (HDL), uric acid, insulin, and fasting plasma C-peptide were assessed before surgery and 1, 3, 6, 12, and 24 months postsurgery.

### Surgical Methods

Laparoscopic RYGB was performed by a single surgeon. For LRYGB, five trocars were used, constructing a 30–50-ml gastric pouch. The length of the biliopancreatic limb was 50–100 cm, and the Roux limb was 100–150 cm. The gastrojejunostomy was created by a staple technique with an anastomosis 1.5–2.0 cm in diameter, and the mesenteric and Petersen defects were closed.

### Endpoints

The primary endpoint was the difference in the rate of T2DM remission between the two groups, with the recommendation from an expert consensus meeting organized by the American Diabetes Association [17]. Complete remission was defined as a fasting glucose level of less than 5.6 mmol/L and a glycated hemoglobin level of less than 6.0% for at least a year without active pharmacologic therapy. Partial remission was defined as a fasting glucose level of 5.6–6.9 mmol/L and a glycated hemoglobin level of less than 6.5% for at least a year without active pharmacologic therapy. Improvement of diabetes was defined as a glycated hemoglobin level of less than 7% for at least a year.

The secondary endpoints were changes from baseline in levels of BMI, waist circumference, FPG, 2hPG, HbA1c%, C-peptide, and levels of plasma TC, TG, HDL, and LDL at 2 years.

### Statistical Analysis

Statistical analysis was performed using SPSS version 17.0 (SPSS Inc., Chicago, IL, USA). Graphs were made using a commercially available software package (GraphPad prism for Windows). Baseline comparisons were performed using chi-square test, paired *t* test, and one-way ANOVA. Continuous variables were expressed as mean ± standard deviation; continuous variables were compared using Student ANOVA for repeated measurement, and two-tailed *p* value < 0.05 was considered statistically significant.

## Results

### Patient Demographics

Seventy patients (39 men and 31 women) with uncontrolled T2DM underwent laparoscopic RYGB between May 2010 and December 2015, and all patients were followed up above 6 months. They were divided into two groups based on the BMI cutoff point of 30 kg/m^2^. Forty-seven patients with BMI < 30 kg/m^2^ were placed in the lower BMI group. (The patients’ demographic data is summarized in Table 1.) Patients with BMI < 30 kg/m^2^ were significantly older, predominantly female and with longer duration of diabetes than those with BMI > 30 kg/m^2^. However, while the lower BMI patients had a trend toward higher FPG (8.91 vs. 8.25 mmol/L; *p* = 0.41), higher HbA1C (8.24 vs. 7.79%; *p* = 0.28) and lower C-peptide level (1.37 vs. 2.01 ng/ml; *p* = 0.14), this was statistically insignificant. The two groups also had similar baseline serum lipid levels, including TC, TG, HDL, and LDL.

**Table 1:** Baseline patient characteristics

Characteristic	BMI < 30 kg/m^2^ (*n* = 47)	BMI > 30 kg/m^2^ (*n* = 23)	*p* value
Age (years)	47.45 ± 8.69	38.74 ± 11.16	0.001*
Male (female)	26 (21)	13 (10)	< 0.001*
Duration of diabetes (years)	5.58 ± 4.40	3.13 ± 3.02	0.009*
Height (cm)	161.83 ± 7.48	163.91 ± 8.60	0.3
Weight (kg)	70.1 ± 9.75	94.56 ± 16.31	< 0.001*
BMI (kg/m^2^)	26.7 ± 2.53	35.17 ± 4.99	< 0.001*
Waist circumference (cm)	92.2 ± 7.67	106.83 ± 9.13	< 0.001*
FPG (mmol/L)	8.91 ± 3.28	8.25 ± 2.52	0.41
2hPG (mmol/L)	16.73 ± 4.7	14.53 ± 4.09	0.06
HbA1c (%)	8.24 ± 2.16	7.79 ± 1.3	0.28
C-peptide (ng/ml)	1.37 ± 1.03	2.01 ± 2.43	0.14
TC (mmol/L)	4.93 ± 1.24	5.21 ± 1.47	0.41
TG (mmol/L)	2.8 ± 2.21	3.41 ± 2.98	0.35
HDL (mmol/L)	1.19 ± 0.65	1.13 ± 0.57	0.71
LDL (mmol/L)	2.75 ± 0.84	2.8 ± 0.81	0.82

*p < 0.05

### Surgical Treatments and Complications

All procedures were successfully performed through the use of laparoscopic techniques. The mean operative time was 125 ± 28 min. The average length of hospital stay was 4.3 ± 1.1 days. There were no early and late mortality. Five patients (7.1%) developed early or late complications, including one patient with gastrojejunal anastomotic leakage, one patient with gastrojejunal anastomotic stenosis, two patients with anastomotic ulcer, and one patient with anemia. All such complications were cured through conservative treatment.

### Glycemic Control

Complete remission of T2DM was found in 28.2% of the BMI < 30 kg/m^2^ group, which was significantly lower than the 57.9% remission rate of the BMI > 30 kg/m^2^ group (*p* = 0.029) (Table 2). Although there was a trend toward less improvement in T2DM remission for lower BMI patients, there was no significant difference in the improvement of T2DM between the BMI < 30 kg/m^2^ group and the BMI > 30 kg/m^2^ group (79.5 vs. 89.5%; *p* = 0.345).

**Table 2:** Remission of T2DM in BMI < 30 kg/m^2^ and BMI > 30 kg/m^2^ groups

Remission of T2DM	BMI < 30 kg/m^2^ (*n* = 39)	BMI > 30 kg/m^2^ (*n* = 19)	*p* value
Complete remission no. (%)	11 (28.2)	11 (57.9)	0.029*
Partial remission no. (%)	8 (20.5)	5 (26.3)	0.619
Improvement no. (%)	31 (79.5)	17 (89.5)	0.345

*p < 0.05

The FPG, 2hPG, and HbA1c% levels were significantly improved in both groups after a month (*p* < 0.05), and they remained essentially stable from the sixth month in both groups. Two years postsurgery, in the BMI < 30 kg/m^2^ group, the mean FPG declined significantly from 8.91 ± 3.28 to 6.42 ± 2.01 (*p* = 0.002), 2hPG declined from 16.73 ± 4.7 to 7.33 ± 3.38 (*p* < 0.001), and HbA1c decreased from 8.24 ± 2.16 to 6.39 ± 0.85 (*p* < 0.001). In the BMI > 30 kg/m^2^ group, the mean FPG decreased significantly from 8.25 ± 2.52 to 5.74 ± 1.01 (*p* = 0.008), 2hPG decreased from 14.53 ± 4.09 to 5.19 ± 1.0 (*p* < 0.001), and HbA1c decreased from 7.79 ± 1.3 to 6.23 ± 0.57 (*p* < 0.001).

**Fig. 1:**
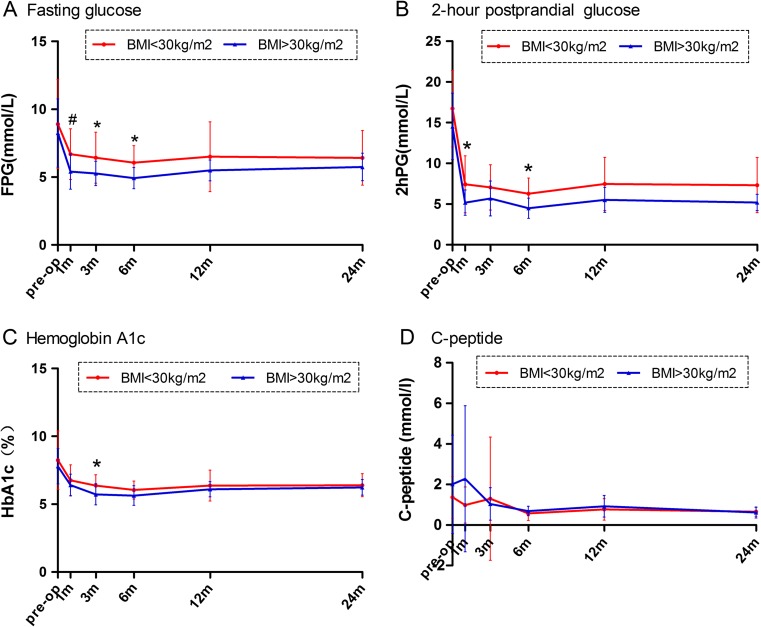
Mean changes in measures of diabetes control from baseline to 2 years between the two groups. **a** Fasting plasma glucose. **b** 2-hour postprandial blood glucose. **c** Glycated hemoglobin A1c. **d** C-peptide. *p < 0.05, ^#^ *p* < 0.01 (BMI < 30 kg/m^2^ vs. BMI > 30 kg/m^2^)

The FPG and 2hPG levels were comparable for the BMI < 30 kg/m^2^ and BMI > 30 kg/m^2^ groups at all time points (Fig. 1a, b). At the 1-, 3-, and 6-month visits postsurgery, the FPG values were much higher for the BMI < 30 kg/m^2^ group than the BMI > 30 kg/m^2^ group (*p* = 0.005, *p* = 0.039, and *p* = 0.014, respectively). At the 1- and 6-month follow-up time points, the 2hPG values were also higher for BMI < 30 kg/m^2^ group (*p* = 0.011 and *p* = 0.013, respectively). At the 3-month visit postsurgery, HbA1c was higher for the BMI < 30 kg/m^2^ group than the BMI > 30 kg/m^2^ group (6.37 vs. 5.72; *p* = 0.017). At 2 years postsurgery, the HbA1c levels were similar in the two groups (6.39 vs. 6.23%; *p* = 0.72) and at all the other follow-up time points (*p* > 0.05) (Fig. 1c). The C-peptide reducing curve tends to be stable and similar from the third month in both groups (Fig. 1d).

### Weight Loss

**Fig. 2:**
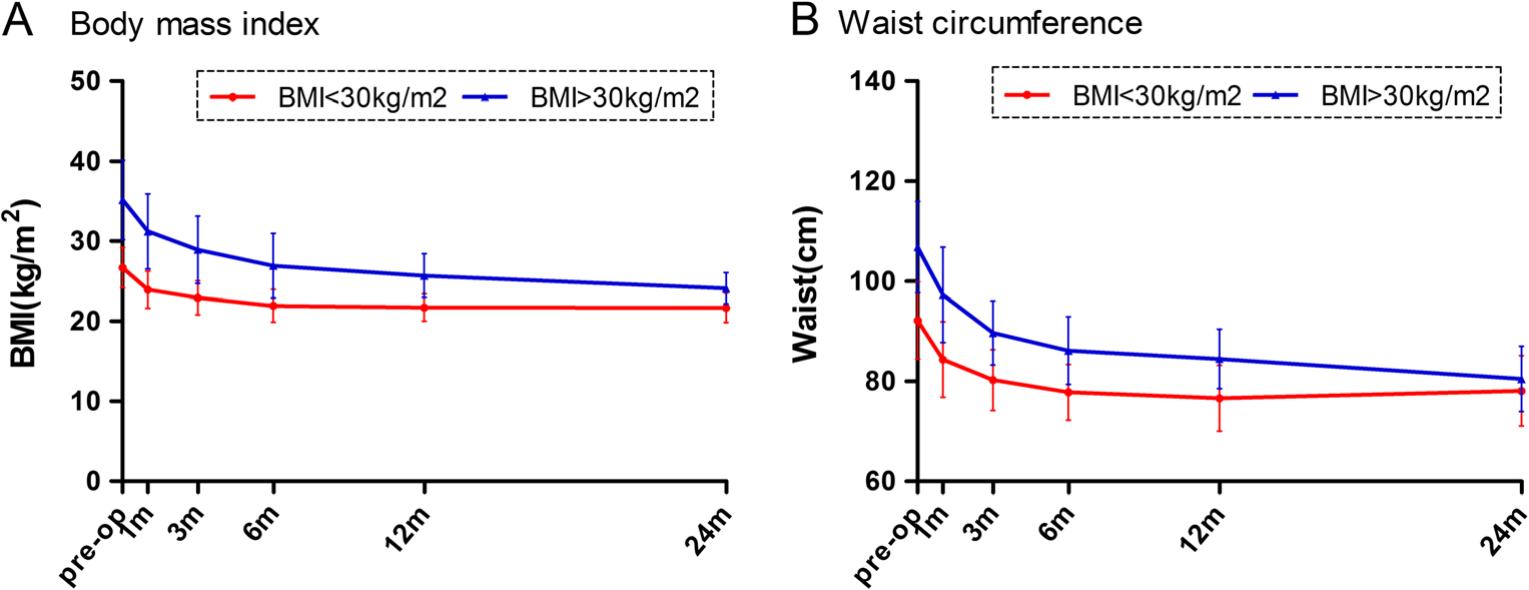
Change in body mass index (**a**) and waist circumference (**b**) from baseline to 2 years between the two groups with different BMI levels. m indicates months after surgery

Patients in both groups showed a significant mean BMI and waist circumference reduction. The preoperative mean BMI was 26.7 kg/m^2^ and significantly decreased to 23.9, 21.9, 21.71, and 21.6 kg/m^2^ at 1, 6, 12, and 24 months postsurgery in the lower BMI group. In the higher group, the mean BMI decreased from 35.2 to 24.1 kg/m^2^. Waist circumference was also significantly reduced in both groups at each visit. The changes in BMI and waist circumferences among the subjects in both groups are shown in Fig. 2a, b. The BMI and waist-reduction curves were similar between the two groups.

### Lipid Profile

**Fig. 3:**
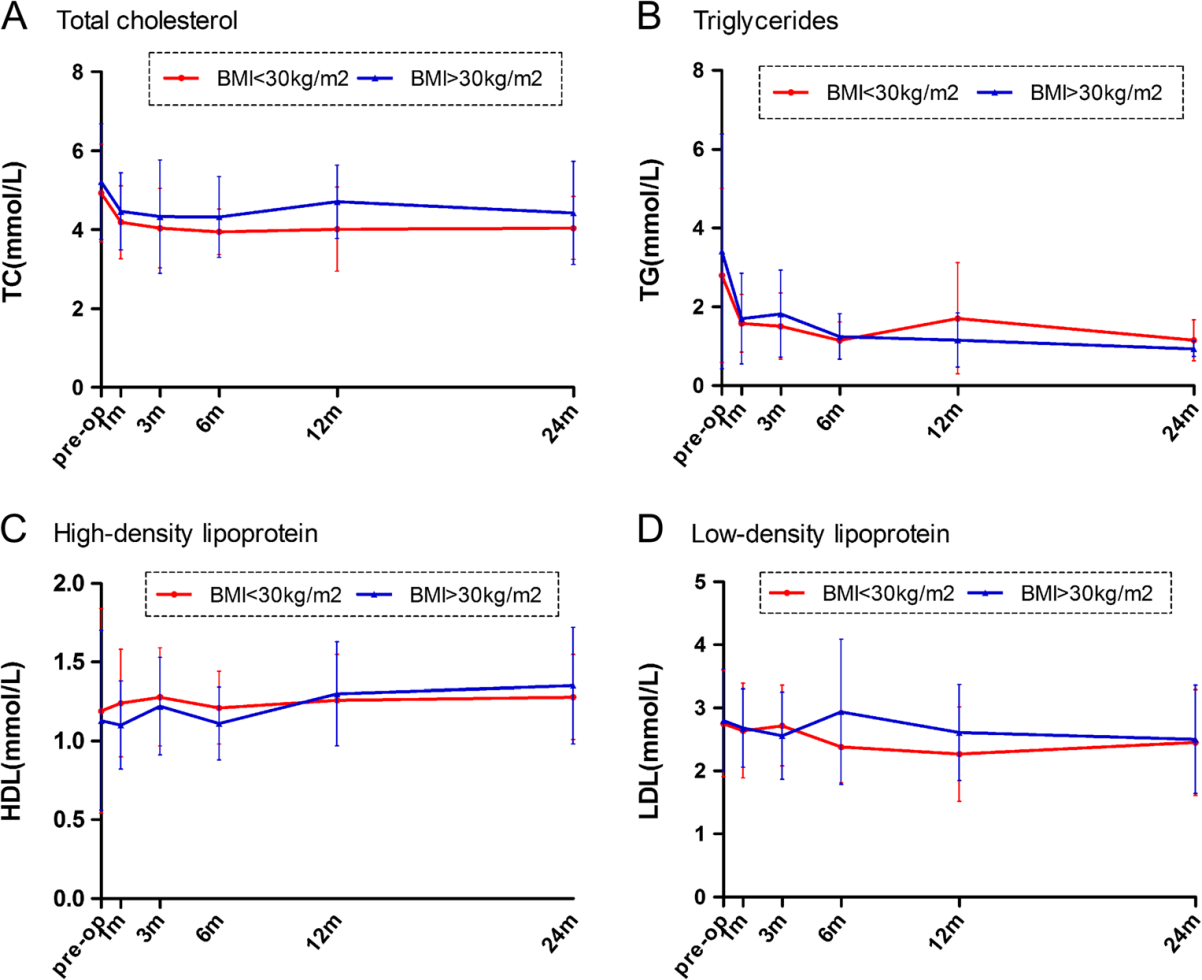
Mean changes in measures of lipid profile from baseline to 2 years between the two groups (BMI < 30 kg/m^2^ vs. BMI > 30 kg/m^2^). **a** Total cholesterol (TC). **b** Triglycerides (TG). **c** High-density lipoprotein (HDL). **d** Low-density lipoprotein (LDL)

Compared to the baseline, postoperative serum triglyceride and HDL in the BMI > 30 kg/m^2^ group and triglyceride in the BMI < 30 kg/m^2^ group were improved (Fig. 3b, c). However, no significant changes were observed in regard to the cholesterol and LDL levels (Fig. 3a, d). The serum levels of cholesterol, triglyceride, HDL, and LDL were similar at each time point for both groups.

## Discussion

The aim of the present study was to compare the effects of RYGB on 47 T2DM patients with BMI < 30 kg/m^2^ and 23 T2DM patients with BMI > 30 kg/m^2^. We wondered whether the lower BMI group would have the same good postoperative response as the higher BMI group. This study confirmed that RYGB was safe and might be useful to treat low BMI T2DM patients, which were consistent with previous reports of non-obese T2DM patients [7, 18–23].

We found that 28.2% of patients in the BMI < 30 kg/m^2^ group had complete remission of T2DM at 12 months postsurgery with an acceptable number of complications. The complete remission are similar to some recent report [12, 24–26], especially those about low BMI patients reported by Dixon et al. [24] and Liang et al. [12], but are lower than those reported by Huang et al. [13]. Dixon et al. [24] reported that glycemic control was achieved in 31 (30%) T2DM patients with BMI < 30 kg/m^2^ at 12 months. Liang et al. [12] found that 25% of patients with BMI < 28 kg/m^2^ had complete remission of T2DM at 12 months. Huang et al. [13] reported that 14 (63.6%) patients with a BMI of 25–35 kg/m^2^ showed T2DM remission when treated by RYGB. Such discrepancies could be explained by the greater severity and longer duration of diabetes in our low BMI population, as well as a stricter definition of remission. Currently, the most important discussion about metabolic surgery should be improvement rather than the complete remission of diabetes, and the ultimate goal of diabetes care should be minimization of end organ damage, particularly vascular complications, instead of reducing the remission of diabetes. Chen Y et al. [5] reported that although complete remission rate can be significantly reduced after long-term follow-up, all patients still had better glycemic control and less number or dosage of medications than before surgery, which might result in reduced vascular complications. A recent RCT and an accompanying editorial highlight that despite the 0% diabetes remission at 5 years, there are also having more than 80% of patients in the surgery group achieved the ADA treatment goal of glycated hemoglobin A1c less than 7.0% and had a greater reduction of diabetes-related complications than patients who received medical treatment [27, 28]. Our study did not show significant difference in the improvement (HbA1c < 7.0%) of T2DM between groups. This means that patients in the BMI < 30 kg/m^2^ group also benefit from gastric bypass surgery even if they have a lower complete remission of diabetes.

The metabolic mechanisms leading to improved glycemic control after metabolic surgery have been partly elucidated. Several mechanisms have been proposed, including increased insulin sensitivity by calorie restriction, subsequent weight loss, decreased secretion of ghrelin and anti-incretin factor, stimulated glucagon-like-peptide-1 (GLP-1) and peptide YY secretion by the rapid transport of nutrient to the distal intestine causes weight loss, increased bile acid, changed gut microbiota, altered most adepokine levels, and probably additional undiscovered effects [29]. It is increasingly clear that the gut plays a major role in glucose homeostasis, regulating both insulin secretion and sensitivity. These theories illustrate that T2DM remission after RYGB was not necessarily associated with weight loss. And further studies have shown that “foregut and hindgut theory” plays an important role in the treatment of T2DM; bypass of the duodenum and jejunum can directly control T2DM without significant weight loss [30, 31]. In this study, the BMI decrease in the lower BMI group was less than that of the higher BMI group, but the efficacy of T2DM treatment was similar and this result is consistent with the previous theory. However, while in some studies, the low weight loss observed in T2DM patients did not affect the capacity of RYGB to induce diabetes remission and substantially improve insulin sensitivity; several recent studies have demonstrated the importance of weight loss on T2DM remission in lower BMI patients. Lee et al. [14] observed that weight loss remained the dominant influence on the remission of T2DM following metabolic surgery in non-obese patients. The Korean study also found that the remission of T2DM was related to postsurgical weight loss of more than 12% [32]. Thus, the weight loss effect on diabetes remission is still controversial and more clinical trials will be necessary before a conclusion can be made.

Numerous studies have demonstrated the high levels of metabolic risk factors at relatively low levels of BMI among Asian population because they have significantly high level of subcutaneous and visceral fat, which corresponds to high risk of cardiovascular and metabolic disease [33, 34]. When it comes to the prevalence of uncontrolled T2DM accompanied by low BMI, there is an urgent need for treatment of T2DM in Asians. Therefore, BMI is regarded as the indication for the appropriateness of metabolic surgery is insufficient [35]. After continuous adjustment and updating, the International Diabetes Federation has recommended that any patient of Asian ethnicity with T2DM should be conditionally eligible for bariatric surgery if his or her BMI is between 27.5 and 32.5 kg/m^2^ and he or she has HbA1c > 7.5% despite fully optimized conventional therapy. Additionally, patients are eligible if their weight is increasing or if other weight responsive co-morbidities are not achieving targets on conventional therapies [36]. Thus, the subjects of this study are mildly obese uncontrolled T2DM with BMI < 30 kg/m^2^.

Although expansion of the RYGB criteria to include lower BMI patients with T2DM is attractive, the issue of the safety needs to be proposed. There was no mortality, no need of reoperation in this study, and the overall morbidity of 7.1% was similar to that of 9 and 10.3% for metabolic surgery in lower BMI patients, as mentioned in two reports by Fried et al. [37] and Huang et al. [13]. In the study by Cohen et al. [38], there was no mortality in a series of 37 patients after RYGB. The combined results of our and others’ study indicate that RYGB in T2DM patients with low BMI can be performed with no mortality and that most of the associated complications can be managed successfully.

Diabetes mellitus is a major risk of cardiovascular disease. Although glycemic control is the primary goal of management, the ultimate objective is to reduce end organ both microvascular (diabetic, nephropathy, neuropathy, retinopathy) and macrovascular (stroke, coronary artery disease, peripheral vascular disease) complications of T2DM. In T2DM patients with obesity, insulin resistance is associated with lipid metabolism. This pattern of lipid abnormalities is thought to be secondary to insulin resistance [39]. In the study by Iaconelli et al. [40], reductions in the levels of triglycerides and LDL cholesterol after biliopancreatic diversion helped normalize insulin sensitivity and to reduce rates of cardiovascular events. Baza et al. [41] found that RYGB induced significant improvement in TG and HDL levels, which might be related to the weight loss and improved insulin resistance. In our study, lipid metabolism was improved in the two groups of patients, although there was no significant statistical difference between those groups. The improvement of the lipid profile might explain the cardiovascular benefits in many studies. Thus, we concluded that RYGB can improve the patients’ cardiovascular risk factors, which may in turn improve their life expectancy in the coming years.

In Asian populations, most T2DM patients have a BMI below 35 kg/m^2^ and impaired islet function during the early stage of disease [2]. Wang GF et al. [42] indicated that old age is associated with lower insulin sensitivity and diminished insulin secretion; duration of diabetes is also known to reflect the residual β-cell mass in T2DM patients. In our study, patients with BMI < 30 kg/m^2^ are significantly older and have longer duration of diabetes. It may indicate that most of low BMI patients with T2DM would have “worse” diabetes. Also, our research has demonstrated that the patients in the BMI < 30 kg/m^2^ group had a lower complete remission rate than the BMI > 30 kg/m^2^ group. Nevertheless, previous studies have shown patients with older age and long diabetes duration were less likely to achieve T2DM remission after bariatric surgery [42, 43]. Therefore, the lower remission rate in the BMI < 30 kg/m^2^ group may be the result of multiple factors; if we exclude the influence of age and diabetes duration, the complete remission of the BMI < 30 kg/m^2^ group may be much higher.

This study had several limitations. First, the case number was relatively small, although fulfilling the sample-size requirement, larger multicenter studies will be necessary in order to confirm our findings. Secondly, although the early operative outcome was satisfactory in terms of safety and glycemic control, long-term data to determine late complications and maintenance of diabetes remission are required. The follow-up period was not long enough to study the final outcome in the two groups of patients.

## Conclusions

In summary, although the complete remission of T2DM in the BMI < 30 kg/m^2^ group is lower than the BMI > 30 kg/m^2^ group, RYGB is still a safe and effective procedure to T2DM with the BMI < 30 kg/m^2^ group. Thus, a longer follow-up period is necessary in order to confirm the long-term effects.
